# Statistical Analysis on Time to Blindness of Glaucoma Patients at Jimma University Specialized Hospital: Application of Accelerated Failure Time Model

**DOI:** 10.1155/2022/9145921

**Published:** 2022-05-14

**Authors:** Meseret Mesfin Bambo, Meskerem Getachew Gebremariam

**Affiliations:** ^1^Department of Statistics, College of Natural and Computational Science, Mizan-Tepi University, Tepi, Ethiopia; ^2^Department of Statistics, College of Natural Science, Jimma University, Jimma, Ethiopia

## Abstract

**Background:**

Glaucoma is one of the most frequent vision-threatening eye diseases. It is frequently associated with excessive intraocular pressure (IOP), which can cause vision loss and damaged optic nerves. The main objective of this study was to model time to blindness of glaucoma patients by using appropriate statistical models. *Study Design*. A Retrospective Community-Based Longitudinal Study design was applied. *Materials and Procedures*. The data were obtained from Ophthalmology Department of JUSH from the period of January 2016 to August 2020. The glaucoma patient's information was extracted from the patient card and 321 samples were included in the study. To discover the factors that affect time to blindness of glaucoma patients', researchers used the Accelerated Failure Time (AFT) model.

**Results:**

81.3 percent of the 321 glaucoma patients were blind. Unilaterally and bilaterally blinded female and male glaucoma patients were 24.92 and 56.38%, respectively. After glaucoma disease was confirmed, the median time to the blindness of both eyes and one eye was 12 months. The multivariable log-logistic accelerated failure-time model fits the glaucoma patient's time to blind dataset well. The result showed that the chance of blindness of glaucoma patients who have absolute stage of glaucoma, medium duration of diagnosis, long duration of diagnosis, and IOP greater than 21 mmHg were high with parameters (*ϕ* = 2.425, *p* value = 0.049, 95% CI [2.249, 2.601]), (*ϕ* = 1.505, *p* value = 0.001, 95% CI [0.228, 0.589]), (*ϕ* = 3.037, *p* value = 0.001, 95% C.I [2.850, 3.22]) and (*ϕ* 0.851, *p* value = 0.034, 95% C.I [0.702, 0.999]), respectively.

**Conclusion:**

The multivariable log-logistic accelerated failure time model evaluates the prognostic factors of time to blindness of glaucoma patients. Under this finding, duration of diagnosis, IOP, and stage of glaucoma were a key determinant factors of time to blindness of glaucoma patients'. Finally, the log-logistic accelerated failure-time model was the best-fitted parametric model based on AIC and BIC values.

## 1. Introduction

Glaucoma is one of the most frequent vision-threatening eye diseases. It is frequently associated with excessive intraocular pressure (IOP) that causes vision loss and possibly blindness if the eye's (optic) nerve fails [1]. The disease has a large global distribution (over 66 million people) and is the second biggest cause of permanent blindness (more than 7 million people bilaterally blind) worldwide [2]. Epidemiologic studies have shown that glaucoma is responsible for 20% of cases of blindness in patients in the Afro-Caribbean population and 6% of patients in a predominantly white population who are defined as blind reported by World Health Organization criteria [3, 4].

Based on visual acuity and visual field criteria, scholars in China on Angle-Closure Glaucoma (ACG) predicted blindness in 6% and 30.1% of patients at presentation with 7% progressing to blindness over a 10-year follow-up. Glaucoma is a neurological illness that causes permanent eyesight loss in many people. Glaucoma is a disease that affects the optic nerve and causes the death of retinal ganglion cells (RGCs) and their axons which causes visual field impairments and eventually vision loss [5, 6]. Glaucoma is a complex visual neuropathy that indicates abnormal intraocular pressure and the development of improved procedures for diagnosing RGC injury but also the finding of medications to cure it [7].

The term “blindness” is defined as severe vision loss in one or both eye's some residual vision. According to the WHO, blindness is defined as having a visual acuity of less than or equal to 0.05 and/or a visual field of less than 10 in a radius around the center fixation in the better eye [8]. Globally, the number of people (aged from 40–80 years) with glaucoma will be expected to be around 111.8 million in 2040 [9]. Glaucoma is a multifactorial aging syndrome marked by the death of retinal ganglion cells and the remodeling of connective tissue at the optic nerve. The excavation of the optic disk and progressive change of the visual field characterize primary open-angle glaucoma (POAG), also known as optic neuropathy [10]. It is a leading cause of visual impairment, affecting 66 million peoples worldwide [11–13].

The majority of the research relied on nonparametric and semiparametric models to determine the time to blindness due to glaucoma. Although semiparametric, nonparametric, and parametric survival models are all useful for analyzing time-to-event data but for some reason, a parametric survival model is selected. The baseline hazard model's distribution is not stated in the semiparametric survival model, but it is assumed to be a well-known distribution in the parametric model [14–16]. Advantages of the parametric model in survival analysis include the distribution of survival time estimate; full maximum likelihood estimate parameters; residuals can represent the difference between observed and estimated values of time; estimated parameters provide clinically meaningful estimates of effect. The following are some of the applications of a parametric model. A parametric model can predict the distribution of survival time, demonstrate the difference between observed and predicted values of time, quantification, model creation using time-dependent factors, complex models in big datasets, and cause-specific or relative survival estimation [17, 18].

## 2. Materials and Methods for Research

The study was conducted at Jimma University's Specialized Hospital. A Retrospective Community-Based Longitudinal Study design was employed to acquire essential information from medical records to meet the study's goal. Confirmed eye patients at Jimma University Specialized Hospitals were the target population of the studies. From January 2016 to August 2020, the medical records of chosen eye ailment patients at Jimma University Specialized Hospital's Ophthalmology Department were examined. During the observation period, each patient's glaucoma-related blindness who has at least one eye was blind was considered an incidence. The response variable in this study was glaucoma patients' survival time to blindness. Patients with glaucoma who survived the study period but were lost to follow-up or died for other reasons were censored.

The explanatory variables are gender (female, male), age, place of residence (rural, urban), diabetes disease (yes, no), duration of diagnosis (short, medium, and long), type of medication (timoglue, diamox, timolol), IOP (normal, not normal), stage of glaucoma (early, moderate, advanced, and absolute), and cup-disc ratio (≤0.7, >0.7). In this paper, the stratification of the stage of glaucoma is based on the American's glaucoma staging systems or new ICD-9 definitions (codes). It allows staging of glaucoma into mild (early), moderate, advanced, and absolute (end-stage glaucoma) based simply on the physician's analysis of the printout of the visual field in the patient's worse eye [19].

The cup-disk ratio cutoff threshold is less than or equal to seven and more than seven in this investigation. The rationale for this case is that a bigger cup-disc ratio >0.7 has a high risk of blindness [20, 21]. Large optic discs with large cups can appear glaucomatous when they have the same neuro-retinal rim area as a smaller disc with a smaller cup [22]. The glaucoma patient's survival time is measured from the commencement of follow-up until the date of blindness (or censor).

### 2.1. Survival Analysis

Survival analysis is the study of occurrences along a well-defined time axis until the occurrence of a specific event or endpoint. Therapeutic recovery, cure time, death time, and blindness time are all examples of events. The time between a certain time and the occurrence of an event is known as survival time. Let *T*  be a random variable representing a survival time, and one of three functions can be used to define the survival time distribution [23–25]. The purpose of survival analysis is to determine the time to event data and the probability that the survival time will be greater or equal to *t*.

(1) $$\begin{array}{c} S\left(t\right)=P\left(T\geq t\right)=1-\;F\left(t\right). \end{array}$$

The probability density function can be used in survival analysis to represent the continuous probability distribution of a random variable such as time. Density functions are shown for the continuous random variable *T*

(2) $$\begin{array}{c} f\left(t\right)=\frac{d}{dt}F\left(t\right)=\frac{F\left(t+h\right)-F\left(t\right)}{h}\;=\frac{f\left(t\right)h}{h}\;. \end{array}$$

Instantaneous failure rate, incidence rate, age-specific failure rate, and conditional failure rate are all terms used to characterize the hazard function. The hazard function expresses the likelihood of an event occurring at any given time *t* (per unit time). Given that one has survived (i.e., has not experienced an event) up to time *t*, the hazard function for continuous random variables is

(3) $$\begin{array}{c} f\left(t\right)=\frac{F\left(t+h\right)-F\left(t\right)}{h}*\;\frac{1}{P\left(T\geq t\right)}=\frac{F\left(t+h\right)-F\left(t\right)}{h}*\;\frac{1}{1-F\left(t\right)}=\frac{f\left(t\right)}{S\left(t\right)}. \end{array}$$

The KM estimator is a nonparametric estimator of the survivor function S (*t*). The KM estimator of the survivorship function (survival probability *S*(*t*) = *P*(*T* ≥ *t*); *S*(*t*) is given by Smith [26].

(4) $$\begin{array}{c} \hat{S}\left(t\right)=\overset{n}{\underset{j<t}{\prod }}{\left[1-\frac{di}{nj}\right]}^{\delta i}, \end{array}$$

where *d*_*i*_ is the number of people who witness the event at the same time  *tj*, *δi* is a tied indicator, and *nj* is the number of persons who have not yet experienced the event and are hence still in danger of doing so at that time. The log-rank test, often known as the Mantel–Cox test, is the most commonly used method for comparing two survival curves and may be easily expanded to comparisons of three or more curves [27].

Because the baseline hazard function is nonparametric, Cox regression is classified as a semiparametric approach, *h*_*o*_(*t*) because the baseline danger is unknown, a distinct parameter is employed for each survival time. The semiparametric model offers a lot of flexibility and is extensively used because the hazard function isn't confined to a certain shape. The hazard ratio (HR) If two people who have distinct covariates *x* and *x*^*∗*^ is

(5) $$\begin{array}{c} \text{HR}=\frac{h_{o}\left(t\right)\exp\left(\beta^{,}x\right)}{h_{o}\left(t\right)\exp\left(\beta^{,}x^{*}\right)}. \end{array}$$

The proportional hazards (PH) model is named after the HR since it is time-independent. The key concept of the Cox PH model is that the repressor coefficients of the hazard function must remain constant across time. Each covariate must confirm the main assumption of this PH model. Despite their advantages over semiparametric models, parametric models are only used in clinical survival research on a limited basis. When the hazard function or relative survival time are the most important markers of association, parametric regression analysis is a good alternative to the often used Cox model. The primary distinction between Cox regulation and AFT is that the baseline hazard function is supposed to follow a specific distribution [28]. An accelerated failure time model (AFT) is a parametric model in the statistical field of survival analysis that offers an alternative to the often used PH models. Covariate multiplies the danger by a constant in a PH model, while it accelerates or decelerates the disease's life cycle by a constant in an AFT model. The model for accelerated failure time is written as follows:

(6) $$\begin{array}{c} \lambda \left(\frac{t}{\theta }\right)=\theta \lambda_{0}\left(\theta t\right), \end{array}$$

where *θ* represents the covariate's combined effect, typically *θ* = exp(−(*βi*^,^*Xi*)).

### 2.2. Inclusion and Exclusion Criteria

Glaucoma is a common disease for the older age person and the risk of blindness due to glaucoma is high around and above the age of 40, the study include glaucoma patients under follow-up at the JUSH whose age was 40 and above, whereas the glaucoma patients who have insufficient information, and whose age was less than 40 were excluded from the study.

### 2.3. Model Comparison

The likelihood ratio test, maximum likelihood, and information criteria were used to compare the models.

## 3. Results

261 (81.3%) of the 321 glaucoma patients were blind, whereas 60 (18.7%) were censored. The glaucoma patients in this study had the shortest and longest diagnostic follow-up durations of 1 month and 60 months, respectively. Glaucoma patients ranging in age from 40 to 84 were included in this study, with a 12-month median survival time for the blind. 107 (33.33%) of the 321 glaucoma patients were female, while 214 (66.67%) were male. 124 (38.63%) of the total patients were from the urban, while the remaining 197 (61.37%) were from the countryside (rural). The age group between 44 and 69 years had contained the highest number of glaucoma patients around 235 (73.21%), the second one is the age group above 70 years had contains 57 (17.75%), and the last category was the age group between 40 and 43 years had contained the lowest number of glaucoma patients around 29 (9.03%), respectively (Table 1).

During the study period, from the total population of glaucoma patients, 112 (34.89%) of the patients had diabetes disease, and 154 (47.97%) had an IOP that was not normal. About 128 (39.88%) and 193 (60.12%) of the patients had a cup-disk ratio less than or equal 0.7 and greater than 0.7, respectively. Similarly, the patients who have early-stage glaucoma, moderate, advanced, and absolute were 32 (9.97%), 152 (47.35%), 72 (22.43%), and 5 (20.24%), respectively. The glaucoma patients who treated by Timoglue were 132 (41.12%), Timolol 92 (28.66%), and Diamox 97 (30.22%). When we see the duration of diagnosis; short time (less than one year) accounted for 123 (38.32%), medium time (1–5 years) around 100 (31.15%), and long time (equal to or greater than 6 years) was accounted for 98 (30.53%) of the total population. Also, the log-rank test showed that the covariates such as duration of diagnosis, stage of glaucoma, and IOP were significant and affect time to blindness of glaucoma patients (Table 2).

**Figure 1:**
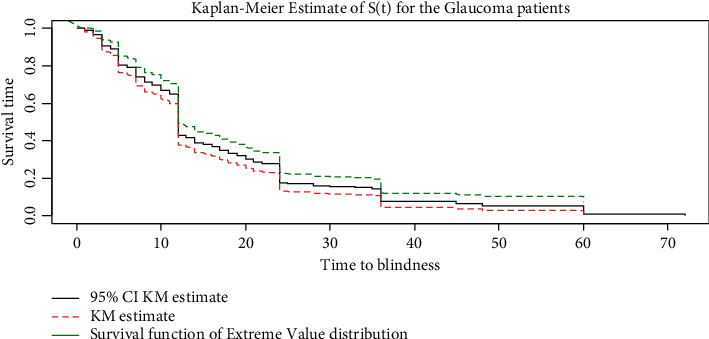
KM plot of glaucoma patients' dataset.

**Figure 2:**
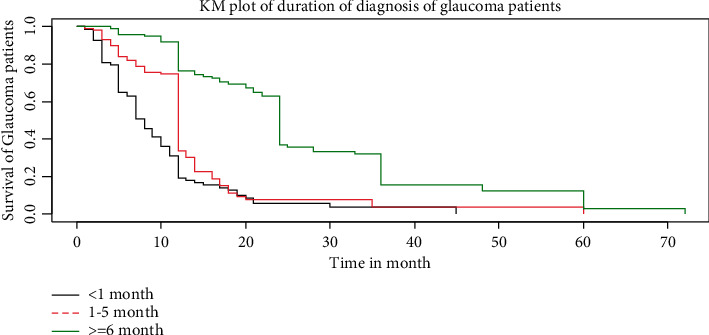
KM plot of duration of diagnosis of glaucoma patients.

The overall estimates of the KM survivor function presented below showed that blindness was higher at the beginning of the follow-up months and it strictly declined in the later months of follow-up (Figure 1). Duration of diagnosis is the significant prognostic factor that hinders survival time of Glaucoma patients. At the starting time of diagnosis, large number of glaucoma patients were on follow-up; however, later the number declines slowly (Figure 2).

### 3.1. Global Test

The duration of diagnosis, stage of glaucoma, and IOP were a statistically significant and affect time to blindness of glaucoma patients at 5% level of significance. For the supplied data, the PH assumption is not met. The *p* value for the overall test was less than 0.05 (*p* value = 0.0041) (Table 3).

### 3.2. The Plot of Schoenfeld Residuals

**Figure 3:**
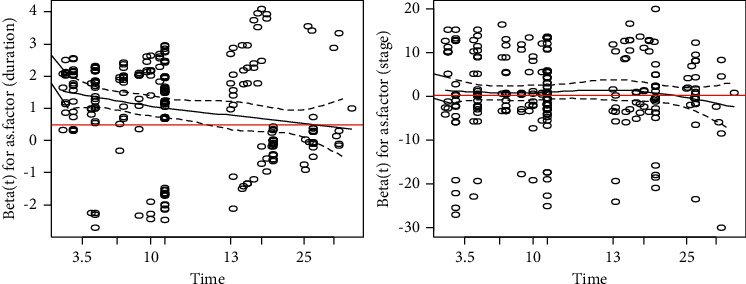
Test of proportionality of hazard ratio.

The plot of Schoenfeld does not support the test proportionality of the Cox-regression model and also the model was inappropriately fit the glaucoma patient's dataset, because of this reason directly proceed to parametric accelerated failure time model (Figure 3).

### 3.3. Model Comparison

The log-logistic hazard function has the lowest AIC (1851.725) and BIC (1878.125) value. This indicates that under the premise of the log-likelihood; log-logistic hazard function is the model chosen to describe time to blindness of glaucoma patients with the maximum likelihood ratio value (Table 4).

### 3.4. Multivariable Log-Logistic Model of Time to Blindness of Glaucoma Patients

Before choosing variables for the model, perform a Univariable analysis on all parameters related to glaucoma patients' time to blindness. The multivariable log-logistic model in this investigation includes parameters that were significant at a 25% level of significance. After that, any potential factors that are significant at a 25% level of significance in the Univariable model but nonsignificant at 5% in the multivariable model were excluded from the multivariable models using the backward selection technique. As a result, IOP, stage of glaucoma and duration of diagnosis were critical factors that determine the time to blindness of glaucoma patients.

The log-logistic model is the most efficient model and best fit to the data, our evidence were AIC and BIC value of log-logistic model. At a 5% level of significance, IOP, stage of glaucoma, and duration of diagnosis were significant and affected the time it took for glaucoma sufferers to go blind. The chance of the blindness of patients with absolute stage of glaucoma had 2.425 times higher when compared to those with early-stage glaucoma, controlling other variables as a constant. Also a risk of blindness of patients with glaucoma whose duration of diagnosis was from one to five months is 2.68 times higher than that of glaucoma patients' whose duration of diagnosis was less than one month's (Table 5).

## 4. Discussion

Glaucoma is the causes of permanent blindness throughout the world, and it is linked to optic nerve damage and visual field loss patterns induced by retinal ganglion cell degeneration [20]. In Ethiopia, glaucoma is the fifth most common cause of blindness and the glaucoma patients tend to aid or goes to health center, after they have become unilaterally or bilaterally blind due to a lack of competent and accessible eye care service, as well as a low degree of public awareness [29]. When examining numerous factors impacting glaucoma patients' time to blindness in study area, the accelerated failure time model can be used to predict and statistically estimate the time to blindness of the glaucoma patients [30]. The blindness in one or both eyes due to glaucoma were taken as the event of interest in this investigation. The predictors variable considered in this study were age, gender, place of residence, type of medication, diabetes disease, stage of glaucoma, duration of diagnosis, IOP, and cup-disk ratio. From this, using the univariable analysis technique, stage of glaucoma, duration of diagnosis, and IOP were the determinant factors of time to blindness of glaucoma patients. And hence, these covariates were used in the multivariable analysis in order to compare the parametric accelerated failure time models. Due to the software packages availability for accelerated failure time models, parametric distributions such as Weibull, Exponential, log-normal, and Log-logistic were used in this study. The AIC and BIC criteria can be used to compare different types of models. The result indicated that, the log-logistic accelerated failure time model was best fit to the glaucoma patient's dataset.

The stage of glaucoma was significant risk factor of time to blindness of glaucoma patients. This conclusion was supported by a study conducted in the United Kingdom [31] and several scholars' research [29, 32, 33]. A patient with moderate, advanced, and absolute stage of glaucoma were at higher risk of blindness than that of the early stages glaucoma. The absolute stage of glaucoma has a huge impact on how long it takes for glaucoma patients to go blind. At this stage, glaucoma is caused by irreversible damage to the optic nerve. Blind patches emerge in your viewing field when this nerve deteriorates. This nerve damage is frequently linked to increased ocular pressure for reasons that clinicians do not fully explain [33–35].

Similarly, the patients who had medium and long duration of diagnosis were reducing the high risk of blindness of glaucoma patients. This finding was consistent with another study (French and Margo, 2010) [36–38]. The result shown that, medium and long duration of diagnosis were reducing the hazard of death or blindness. Glaucoma pain should be reported to an eye specialist on a regular basis so that the condition can be detected and treated before a long-term visual loss occurs. Once eyesight has been lost, it is impossible to recover it. On the other hand, properly identifying glaucoma patients and reducing eye pressure can help prevent vision loss, and people with glaucoma can preserve their vision if they follow their treatment plan and have regular eye exams [39]. Additionally, this finding indicated that, IOP had a significant risk factor of time to blindness of glaucoma patients (Table 5). This discovery was supported by the scholar conducted by (Oliver et al.) [29, 40, 41]. Glaucoma is a condition in which the optic nerve is damaged by high eye pressure and resulting in vision loss or blindness. Monitoring IOP is critical thing for detecting blindness due to glaucoma. Another key conclusion of this study is that, it used Schoenfeld residual to determine the significant model for the investigation. For each individual experiencing an event at a given time, the Schoenfeld residuals are generated for all covariates. These are the disparities between that individual's covariate values at the event time and the risk-weighted average of all individuals at risk. The study also looks at survival data rather than cross-sectional data. When it comes to describing a patient's entire medical history, cross-sectional data isn't as good as survival data. Collecting survival data from incident cases normally takes a significant study time in order to acquire enough events for useable analysis. When the frequency of disease remains constant across time, cross-sectional data, on the other hand, produces length-biased survival results (Wang et al.) [42, 43]. Lastly after the study was recovered awareness should be given to the community to reduce the burden of glaucoma.

### 4.1. Strengths and Weaknesses of the Study

The median time to blindness of glaucoma patients has not been determined at Jimma University specialized hospital, and the median time to blindness in the hospital is unknown. This study is noteworthy since it looks at both parametric and nonparametric survival analyses and examined the median time to blindness of glaucoma patients at JUSH. Also another brand-news in this study was on type of medication, timoglue medication is only available at Jimma University's specialized hospital, and researchers have yet to discover it. This treatment distinguishes our discovery from earlier ones. The study's fundamental problem is that it only considers factors that influence time to blindness; however, time to cure from glaucoma disease is not considered in this study.

## 5. Conclusion

The majority of glaucoma patients in this trial were blinded. This finding indicates that predictor variables such as stage of glaucoma, duration of diagnosis, and IOP were significantly affect the time to blindness of glaucoma patients. The accelerated failure time model was used, to identify the risk factors of time to blindness of glaucoma patients'. The log-logistic accelerated failure time model was performed better in terms of overall model parsimony and quality of fit, as evidenced by its lower AIC and BIC values.

## Figures and Tables

**Table 1:** Group differences in some sociodemographic factors and glaucoma patient survival patterns at Jimma University specialized hospital, 2016–2020 (*n* = 321)).

Covariates	Category	Number of censored	Percentage censored	Number of events	Percentage event	Log-rank test	
						Chq	*p* value
Age	40–43	8	2.49	21	6.54	1.6	0.5
	44–69	42	13.08	193	60.12		
	≥70	10	3.11	47	14.64		
Gender	Female	27	8.41	80	24.92	0.3	0.6
	Male	33	10.28	181	56.38		
Place of residence	Rural	35	10.90	162	50.46	2.4	0.1
	Urban	25	7.78	99	30.84		

Source: Jimma University Specialized Hospitals, Ethiopia; from January 1, 2016, to August 30, 2020.

**Table 2:** Clinical variables of glaucoma patients' at Jimma University Specialized Hospital from 2016 to 2020 (*n* = 321).

Covariates	Category	Number of censored	Percentage of censored	Number of events	Percentage of event	Log-rank test	
						Chq	*p* value
Duration of diagnosis	Short (<1 year)	26	8.10	97	30.21	11.0	0.001
	Medium (1–5 years)	20	6.23	80	24.92		
	Long (≥6 years)	14	4.32	84	26.17		
Type of medication	Timoglue	24	7.48	108	33.64	3.90	0.100
	Timolol	17	5.29	75	23.36		
	Diamox	19	5.91	78	24.29		
Stage of glaucoma	Early	11	3.43	21	6.540	13.2	0.004
	Moderate	20	6.23	132	41.12		
	Advanced	29	9.03	43	13.39		
	Absolute	0	-	65	20.25		
Cup-disk ratio	≤0.7	34	10.59	94	29.28	2.30	0.100
	>0.7	26	8.11	167	52.11		
IOP	Normal	21	6.54	146	45.48	0.60	0.012
	Not normal	39	12.15	115	35.83		
Diabetics	No	42	13.11	167	52.02	1.90	0.200
	Yes	18	5.61	94	29.28		

Source: Jimma University Specialized Hospitals, Ethiopia; from January 1, 2016, to August 30, 2020.

**Table 3:** Formal statistical test of glaucoma patients' survival patterns at Jimma University Specialized Hospital from 2016 to 2020 (*n* = 321).

Covariates	Chq	Def.	*p* value
Age group	0.3780	2	0.8270
Stage of glaucoma	6.2000	3	0.0220
Gender	0.2790	1	0.5970
Type of medicine	5.1420	2	0.0764
IOP	1.0440	1	0.0318
Duration of diagnosis	14.020	2	0.0009
Cup-disk ratio	0.9530	1	0.3289
Diabetes	2.6070	1	0.1064
GLOBAL	37.016	13	0.0041

Source: Jimma University Specialized Hospitals, Ethiopia; from January 1, 2016, to August 30, 2020.

**Table 4:** Comparison of models for glaucoma patient's dataset.

No	Model	Log-likelihood	AIC	BIC
1	Weibull	−932.9	1879.749	1906.149
2	Exponential	−922.8	1970.603	1993.232
3	Log-normal	−979.3	1859.501	1885.901
4	Log-logistic	−918.9	1851.725	1878.125

Source: Jimma University Specialized Hospitals, Ethiopia; from January 1, 2016, to August 30, 2020. AIC = Akaike's information criteria; BIC = Bayesian information criteria.

**Table 5:** Multivariable analysis of glaucoma patient's dataset at Jimma University specialized hospital, 2016–2020 (*n* = 321).

Covariates	Category	The semiparametric model of glaucoma patients at Jimma University Specialized Hospital, 2016–2020					The log-logistic accelerated failure time model of glaucoma patients at Jimma University Specialized Hospital, 2016–2020				
		B	Exp (*β*)	St. Err(*β*)	*p* value	95% CI for Exp (*β*)	Β	Exp (*β*)	St. Err (*β*)	*p* value	95% CI for Exp (*β*)
Age	40–43 (ref)										
	44–69	0.388	1.475	0.2384	0.1020	[0.924–2.353]	−0.229	0.794	0.133	0.084	[0.533–1.054]
	≥70	0.114	1.122	0.2752	0.6760	[0.678–2.011]	−0.039	0.961	0.154	0.797	[0.659–1.262]
Gender	Female (ref)										
	Male	0.182	1.199	0.142	0.2039	[0.906–1.586]	−0.086	0.918	0.081	0.293	[0.759–1.076]
Stage of glaucoma	Early (ref)										
	Moderate	0.337	1.399	0.250	0.1780	[0.921–2.461]	−0.109	0.896	0.143	0.443	[0.615–1.176]
	Advanced	0.041	1.041	0.276	0.8840	[0.602–1.778]	0.040	1.041	0.154	0.794	[0.739–1.342]
	Absolute	0.554	1.739	0.270	0.0400	[1.063–3.077]	0.886	2.425	0.090	0.049	[2.249–2.601]
Duration of diagnosis	<1*month*										
	1–5 months	−0.558	0.572	0.163	0.0006	[0.426–0.812]	0.409	1.505	0.092	0.001	[0.228–0.589]
	≥6 months	−1.592	0.203	0.176	0.0010	[0.141–0.279]	1.111	3.037	0.095	0.001	[2.850–3.22]
Cup-disk ratio	≤0.7 (ref)										
	>0.7	0.133	1.141	0.1353	0.3260	[0.885–1.510]	0.039	1.039	0.079	0.623	[0.884–1.193]
IOP	Normal (ref)										
	Not normal	0.133	1.143	0.134	0.320	[0.899–1.535]	−0.161	0.851	0.076	0.034	[0.702–0.999]
Type of medication	Timoglue (ref)										
	Timolol	−0.039	0.961	0.158	0.8030	[−0.73–1.371]	0.0886	1.093	0.090	0.326	[0.916–1.269]
	Diamox	0.249	1.282	0.152	0.1030	[0.97–1.774]	−0.130	0.877	0.092	0.159	[0.696–1.057]
Diabetics	No (ref)										
	Yes	0.039	1.039	0.138	0.7780	0.780–1.353	−0.039	0.961	0.079	0.619	[0.806–1.115]

Source: Jimma University Specialized Hospitals, Ethiopia; from January 1, 2016, to August 30, 2020, ref = reference group.

## Data Availability

The datasets used in this study are available from the corresponding author on reasonable request.

## Abbreviations

AFT: Accelerated failure time
JUSH: Jimma University Specialized Hospitals
IOP: Intraocular pressure
POAG: Primary open-angle glaucoma
AIC: *Akaike's* information criteria
BIC: Bayesian information criteria
CI: Confidence interval.

## References

[1] Quaranta L., Riva I., Gerardi C., Oddone F., Floriano I., Konstas A. G. P. (2016). Quality of life in glaucoma: a review of the literature. *Advances in Therapy*.

[2] Katz L. J., Steinmann W. C., Kabir A., Molineaux J., Wizov S. S., Marcellino G. (2012). Selective laser trabeculoplasty versus medical therapy as initial treatment of glaucoma. *Journal of Glaucoma*.

[3] Foran S., Wang J. J., Mitchell P. (2003). Causes of visual impairment in two older population cross-sections: the blue mountains eye study. *Ophthalmic Epidemiology*.

[4] Leske M. C., Wu S.-Y., Hyman L. (2004). Four-year incidence of visual impairment. *Ophthalmology*.

[5] Quek D. T. L., Koh V. T., Tan G. S., Perera S. A., Wong T. T., Aung T. (2011). Blindness and long-term progression of visual field defects in Chinese patients with primary angle-closure glaucoma. *American Journal of Ophthalmology*.

[6] Rao A. (2012). Blindness and long-term progression of visual field defects in Chinese patients with primary angle-closure glaucoma. *American Journal of Ophthalmology*.

[7] Almasieh M., Levin L. A. (2017). Neuroprotection in Glaucoma: Animal Models and Clinical Trials.

[8] Orton S. T. (1925). “Word-blindness” in school children. *Archives of Neurology And Psychiatry*.

[9] Tham Y.-C., Li X., Wong T. Y., Quigley H. A., Aung T., Cheng C.-Y. (2014). Global prevalence of glaucoma and projections of glaucoma burden through 2040: a systematic review and meta-analysis. *Ophthalmology*.

[10] Asemu M. T., Yesuf K. M., Asnaqew Y. T. (2019). Survival analysis of glaucoma patients until blindness: the case of university of gondar comprehensive specialized hospital, gondar, Ethiopia. *Turkiye Klinikleri Journal of Biostatistics*.

[11] Desai A., Patel D., Sapovadia A., Mehta P., Brahmbhatt J. (2018). A study of relation between primary open angle glaucoma and type II diabetes mellitus. *International Journal of Research in Medical Sciences*.

[12] Meer E., Qin V. L., Gudiseva H. V. (2021). LMX1B locus associated with low-risk baseline glaucomatous features in the POAAGG study. *Genes*.

[13] Cai J.-C., Chen Y.-L., Cao Y.-H., Babenko A., Chen X. (2022). Numerical study of aqueous humor flow and iris deformation with pupillary block and the efficacy of laser peripheral iridotomy. *Clinical Biomechanics*.

[14] Shiau C.-Y., Sneed P. K., Shu H.-K. G. (1997). Radiosurgery for brain metastases: relationship of dose and pattern of enhancement to local control. *International Journal of Radiation Oncology, Biology, Physics*.

[15] Boccardo F., Rubagotti A., Guglielmini P. (2006). Switching to anastrozole versus continued tamoxifen treatment of early breast cancer. Updated results of the Italian tamoxifen anastrozole (ITA) trial. *Annals of Oncology*.

[16] Castro J. R., Linstadt D. E., Bahary J.-P. (1994). Experience in charged particle irradiation of tumors of the skull base: 1977–1992. *International Journal of Radiation Oncology, Biology, Physics*.

[17] Gelber R. D., Goldhirsch A., Cole B. F., I.B.C.S. Group (1993). Parametric extrapolation of survival estimates with applications to quality of life evaluation of treatments. *Controlled Clinical Trials*.

[18] Achilonu O. J. (2017). Modelling graft survival after kidney transplantation using semi-parametric and parametric survival models.

[19] Wensor M. D., McCarty C. A., Stanislavsky Y. L., Livingston P. M., Taylor H. R. (1998). The prevalence of glaucoma in the melbourne visual impairment project. *Ophthalmology*.

[20] Davis B. M., Crawley L., Pahlitzsch M., Javaid F., Cordeiro M. F. (2016). Glaucoma: the retina and beyond. *Acta Neuropathologica*.

[21] Bendel R. E., Kaplan J., Heckman M., Fredrickson P. A., Lin S.-C. (2008). Prevalence of glaucoma in patients with obstructive sleep apnoea-a cross-sectional case-series. *Eye*.

[22] Song X., Song K., Chen Y.

[23] Schlattmann P. (2009). *Medical Applications of Finite Mixture Models*.

[24] Veneti Z., Zabalou S., Papafotiou G. (2012). Loss of reproductive parasitism following transfer of male-killing Wolbachia to Drosophila melanogaster and Drosophila simulans. *Heredity*.

[25] Machin D., Cheung Y. B., Parmar M. (2006). *Survival Analysis: A Practical Approach*.

[26] Smith T., Smith B. (2003). *SAS Conference Proceedings: SAS Users Group International*.

[27] Lambert P., Collett D., Kimber A., Johnson R. (2004). Parametric accelerated failure time models with random effects and an application to kidney transplant survival. *Statistics in Medicine*.

[28] Pugh M. G., Robins J., Lipsitz S., Harrington D. (1993).

[29] Oliver J. E., Hattenhauer M. G., Herman D. (2002). Blindness and glaucoma: a comparison of patients progressing to blindness from glaucoma with patients maintaining vision. *American Journal of Ophthalmology*.

[30] Fellman R., Mattox C., Ross K., Vicchrilli S. (2011). *Eye*.

[31] Bruce D., Eshun V. M. (2017). Psychological experience of clients diagnosed with glaucoma in two selected eye clinics in Accra, Ghana. *International Journal of Regulation and Governance*.

[32] Sihota R., Angmo D., Ramaswamy D., Dada T. (2018). Simplifying “target” intraocular pressure for different stages of primary open-angle glaucoma and primary angle-closure glaucoma. *Indian Journal of Ophthalmology*.

[33] Fraser S., Bunce C., Wormald R., Brunner E. (2001). Deprivation and late presentation of glaucoma: case-control study. *BMJ*.

[34] Saba T., Bokhari S. T. F., Sharif M., Yasmin M., Raza M. (2018). Fundus image classification methods for the detection of glaucoma: a review. *Microscopy Research and Technique*.

[35] Thakur N., Juneja M. (2018). Survey on segmentation and classification approaches of optic cup and optic disc for diagnosis of glaucoma. *Biomedical Signal Processing and Control*.

[36] Islam M. M., Buznyk O., Reddy J. C. (2018). Biomaterials-enabled cornea regeneration in patients at high risk for rejection of donor tissue transplantation. *NPJ Regenerative Medicine*.

[37] Barbosa-Breda J., Himmelreich U., Ghesquière B., Rocha-Sousa A., Stalmans I. (2018). Clinical metabolomics and glaucoma. *Ophthalmic Research*.

[38] Kyari F., Adekoya B., Abdull M. M., Mohammed A. S., Garba F. (2018). The current status of Glaucoma and Glaucoma care in Sub-Saharan Africa. *Asia-Pacific Journal of Ophthalmology*.

[39] C.N.-T.G.S. Group (1998). The effectiveness of intraocular pressure reduction in the treatment of normal-tension glaucoma. Collaborative normal-tension Glaucoma study group. *American Journal of Ophthalmology*.

[40] Kastner A., King A. J. (2020). Advanced glaucoma at diagnosis: current perspectives. *Eye*.

[41] Wurster P., Harris A., Gonzalez A. C. (2020). Risk factors for open-angle glaucoma in persons of Latin American descent. *Journal of Glaucoma*.

[42] Wang M.-C. (1991). Nonparametric estimation from cross-sectional survival data. *Journal of the American Statistical Association*.

[43] Asgharian M., M’Lan C. E., Wolfson D. B. (2002). Length-biased sampling with right censoring. *Journal of the American Statistical Association*.

